# Adult presentation of locked ‘congenital’ trigger thumb: A case report

**DOI:** 10.1016/j.ijscr.2023.108827

**Published:** 2023-09-21

**Authors:** Marvin Man Ting Chung

**Affiliations:** Department of Orthopaedics & Traumatology, The University of Hong Kong, Hong Kong

**Keywords:** Trigger thumb, Congenital, Paediatric, Trigger finger, Hand surgery

## Abstract

**Introduction and importance:**

Although ‘congenital’ or paediatric trigger thumb is commonly seen in the paediatric age group, adult presentation is very rare. However it is crucial to acknowledge the occurrence of unusual manifestations of paediatric trigger thumbs, since paediatric trigger thumbs are considered a separate disease entity compared to the commonly seen stenosing tenosynovitis in adults.

**Case presentation:**

We report a case of a 18-year-old young lady presenting with atraumatic locked trigger thumb, which was successfully treated surgically with intra-operative finding confirming abnormally thickened flexor pollicis longus tendon signifying a paediatric trigger thumb pathology.

**Clinical discussion:**

Adult and paediatric trigger thumbs have different aetiology, with thickened A1 pulley and tendon sheath being the culprit in adults, whereas in paediatric thumbs thickened tendon nodules (Notta's node) are usually the causes of triggering.

**Conclusion:**

This uncommon presentation in this case report is atypical in the age group presentation of paediatric trigger thumb, and should be distinguished from the usual trigger thumb pathology in adults. Although a transient period of extension lag in the early post-operative period may be evident, it can still be successfully treated with surgical release.

## Highlights

• It is important to recognize atypical presentation of paediatric trigger thumb even in the adult age group.
• The underlying pathology of paediatric trigger thumb is different from adult trigger thumb.
• Surgical release of A1 pulley is still an effective treatment despite the long delay.

## 1 Introduction

Congenital trigger thumb, or paediatric trigger thumb, is a different disease entity from adult trigger thumb and usually present at the age of 2. Paediatric trigger thumb affects less than 0.05 % of children and often presents with a thumb locked in flexed posture instead of clicking symptoms and pain [1]. The underlying pathology is at the flexor pollicis longus tendon, with an abnormally thickened tendon nodule (also known as Notta's node) at the volar thumb base level [2]; rather than a thickened A1 pulley and stenosis of tendon sheath causing entrapment of a relatively normal flexor tendon as in the case of adult trigger thumb [3]. Freiberg et al. has subclassified adult trigger digit into nodular and diffuse types, where nodular trigger digits are more likely to resolve with steroid injection [4]. However, the nodular swelling they described corresponded to nodular A1 pulley thickening, and more extensive less-defined swelling respectively, which are both distinct from the primary pathology in paediatric trigger digit where the A1 pulley mostly remains normal.

We report an uncommon case of a young adult lady who presented late with a locked ‘congenital’ trigger thumb at 18 years old, with the intra-operative finding of normal A1 pulley but abnormally thickened flexor pollicis longus tendon, which was successfully treated with surgical release resulting in normal thumb function. This work has been reported in line with the SCARE criteria [5].

## 2 Case presentation

A right-handed 18 year-old female patient, working as a part-time waitress in the hotel industry, presented with locked interphalangeal joint (IPJ) of the right thumb for 6 months. She enjoyed good past medical health and had no background of ligamentous or joint diseases. She first noticed clicking sensation in her right thumb since childhood for more than 10 years and did not remember the exact onset. She denied significant trauma or injury to the right hand otherwise. She did not seek medical advice and enjoyed the clicking sensation without functional deficit. However, she noticed the right thumb IPJ became locked suddenly after the usual clicking one day without trauma, and since then she could not actively or passively extend the IPJ. She thought the situation would gradually improve and delayed medical consultation until 6 months later when she noticed that the locked thumb remained the same.

**Fig. 1:**
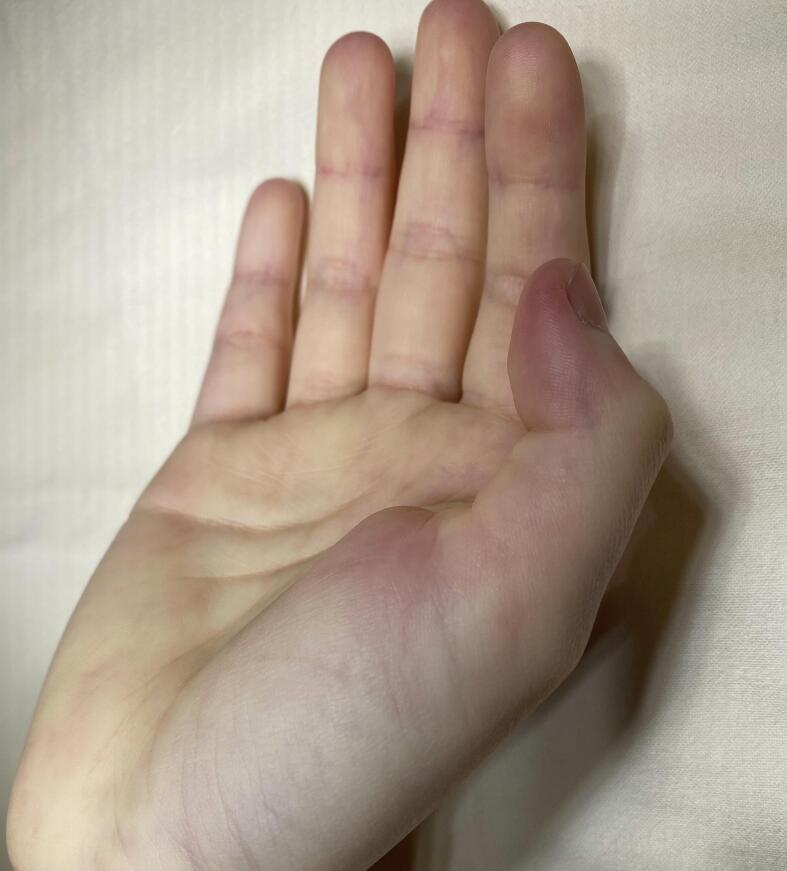
Locked trigger thumb showing interphalangeal joint of right thumb locked at 55 degrees of flexion.

On physical examination, there was a nodular thickening over the volar metacarpophalangeal joint (MCPJ) of the right thumb, which was non-tender on palpation. The IPJ was locked at 55 degrees of flexion, with no further passive extension possible even with the MCPJ placed in flexion (Fig. 1). Extensor pollicis longus action was palpable and was still in function.

**Fig. 2:**
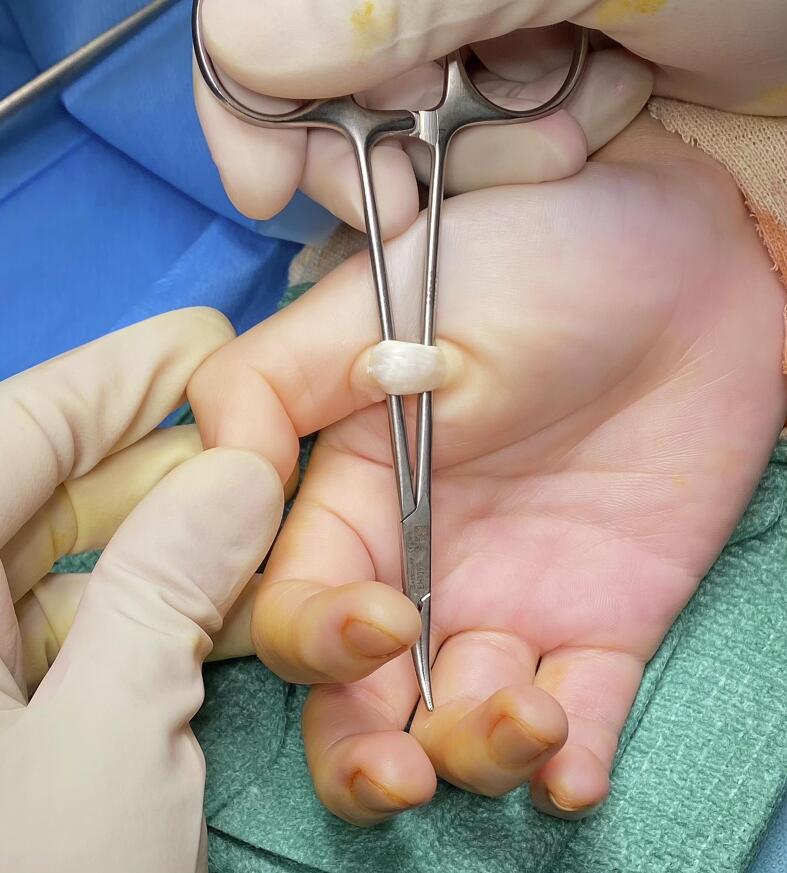
Intra-operative finding of abnormal thickening of flexor pollicis longus tendon.

In view of the delayed presentation with prolonged period of locked thumb, she was directly scheduled to have open surgical release of the right trigger thumb. The operation was performed under local anaesthesia using 1 % lignocaine. A transverse incision was made over the volar MCPJ skin crease. Digital neurovascular bundles were protected with retractors. The A1 pulley was divided longitudinally with the flexor pollicis longus tendon freed and slinged out to confirm complete release. The A1 pulley was noted to be normal looking without thickening. However, whitish fusiform abnormal hard thickening of the flexor pollicis longus tendon was noted underneath and immediately proximal to the A1 pulley (Fig. 2). Full passive range of motion of the thumb IPJ and MCPJ was attained intra-operatively without clicking. Active movement by the patient intra-operatively also confirmed no residual triggering motion but there was an extension lag of 20 degrees due to attenuation of extensor mechanism. Total operative time was 12 min.

Post-operatively she was referred to the physiotherapists and occupational therapists for further rehabilitation. Stitches were removed on post-operative day 14 as usual. There were no complications from the surgery including infection, wound complication, neurovascular injury or recurrent triggering symptoms.

**Fig. 3:**
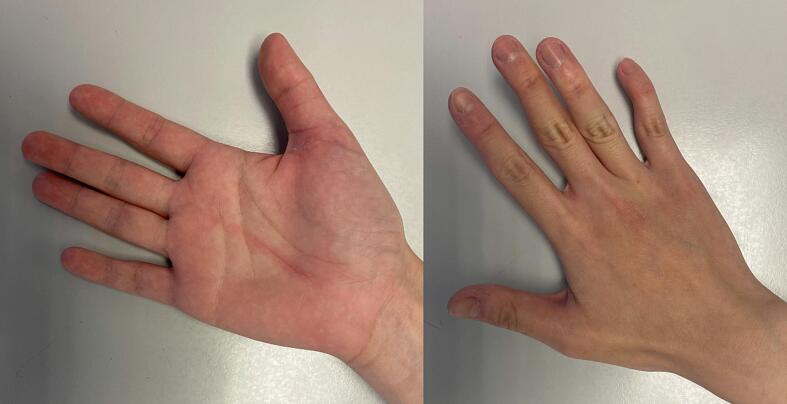
Post-operative results at 5 weeks after operation, showing resolution of extension lag in interphalangeal joint of right thumb.

**Fig. 4:**
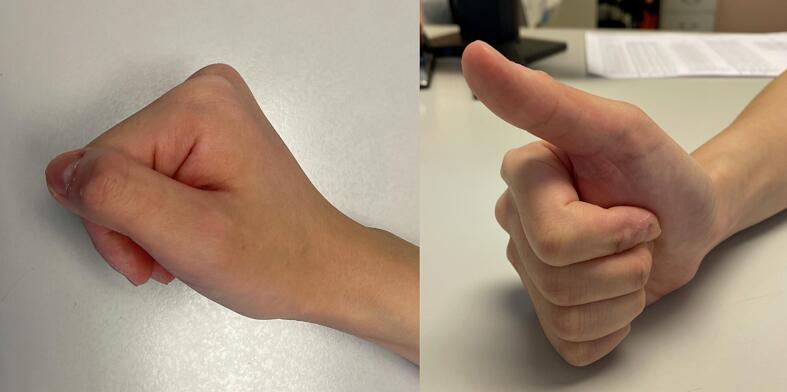
Satisfactory key pinch and active individual control of right thumb.

She was given a course of rehabilitation therapy including scar massage, mobilization and strengthening exercise. At post-operative 2 weeks there was still an extension lag of 20 degrees, limited by wound pain at numeric pain rating scale of 4 out of 10, which subsided later at post-operative 5 weeks (Fig. 3, Fig. 4). Her power grip improved from 16 kg at post-operative 3 weeks to 24 kg at post-operative 6 weeks, whereas the pinch grip improved from 2 kg (120 % of contralateral hand) at post-operative 3 weeks to 5.5 kg (122 % of contralateral hand) at post-operative 6 weeks. There was no limitation in her activities of daily living and work.

## 3 Discussion

Trigger thumb or trigger digit in adolescence are rare, with only isolated reports secondary to trauma [6], overuse such as fencing [7] and excessive texting [8], or other flexor tendon pathologies [9]. However, an acute-on-chronic presentation of ‘congenital’ trigger thumb with locked IPJ during late adolescence or adulthood, together with the classical intra-operative finding of normal A1 pulley and abnormally thickened flexor pollicis longus tendon has not been reported in the literature so far.

The term of ‘congenital’ trigger thumb, however, is considered to be a misnomer by some as it has been reported to be an acquired condition [10] and therefore paediatric trigger thumb is considered to be a more appropriate term. Nevertheless, the pathology usually presents between 6 months to 2 years of age, and would spontaneously resolve in up to half of the cases [1]. Surgical treatment in paediatric trigger thumb, even in the case of delayed diagnosis or treatment, provided satisfactory outcome with release of A1 pulley [11]. There would usually be a period of extension lag in the early post-operative period but was shown to improve in all cases within 1 to 8 weeks in a case series of patients ranging from age 5 to 12 at surgery [12]. However the results of more than 10 years of delay in diagnosis and treatment as in this case at adulthood or late adolescence has not been reported in the literature.

In this case the intra-operative findings were compatible with the paediatric trigger thumb despite the adult presentation. Her late presentation of locked thumb could be triggered by overuse due to her work nature as a waitress, although no definite direct cause was found to be associated with this acute-on-chronic presentation. In retrospect, pre-operative imaging with ultrasound and magnetic resonance imaging scans might provide radiological information regarding the thickened tendon, but performing such investigation is not a routine in majority of hand surgery centres around the world as it does not affect the clinical decision of surgical treatment, method of surgical release with longitudinal division of A1 pulley, or the subsequent rehabilitation.

## 4 Conclusion

This case report illustrates that conservatively-treated or symptom-free paediatric trigger thumb during childhood might remain symptomatic into adulthood, or present acutely with locked IPJ, but surgical release of A1 pulley is still an effective treatment despite the long delay.

## Informed consent

Written informed consent was obtained from the patient for publication of this case report and accompanying images. A copy of the written consent is available for review by the Editor-in-Chief of this journal on request.

## Declaration of competing interest

The author(s) declare that they have no competing interests. No funding or sponsors were involved in this study.
